# Targeting the hedgehog pathway in MET mutation cancers and its effects on cells associated with cancer development

**DOI:** 10.1186/s12964-023-01333-8

**Published:** 2023-11-02

**Authors:** Yifan Du, Huimin Sun, Zhiyuan Shi, Xiuyuan Sui, Bin Liu, Zeyuan Zheng, Yankuo Liu, Zuodong Xuan, Min Zhong, Meiling Fu, Yang Bai, Qian Zhang, Chen Shao

**Affiliations:** 1 Department of Urology, Xiang’an Hospital of Xiamen University, School of Medicine, Xiamen University, Xiamen, 361101 China; 2 Central Laboratory, Xiang’an Hospital of Xiamen University, School of Medicine, Xiamen University, Xiamen, 361101 China; 3 Department of Endocrinology, Xiang’an Hospital of Xiamen University, School of Medicine, Xiamen University, Xiamen, 361000 China

**Keywords:** MET mutation, Cancer, Hh pathway, Cancer-associated fibroblasts (CAFs), HGF/c-MET axis

## Abstract

The mutation of *MET* plays a crucial role in the initiation of cancer, while the Hedgehog (Hh) pathway also plays a significant role in cell differentiation and the maintenance of tumor stem cells. Conventional chemotherapy drugs are primarily designed to target the majority of cell populations within tumors rather than tumor stem cells. Consequently, after a brief period of remission, tumors often relapse. Moreover, the exclusive targeting of tumor stemness cell disregards the potential for other tumor cells to regain stemness and acquire drug resistance. As a result, current drugs that solely target the HGF/c-MET axis and the Hh pathway demonstrate only moderate efficacy in specific types of cancer. Mounting evidence indicates that these two pathways not only play important roles in cancer but also exert significant influence on the development of resistance to single-target therapies through the secretion of their own ligands. In this comprehensive review, we analyze and compare the potential impact of the Hh pathway on the tumor microenvironment (TME) in HGF/c-MET-driven tumor models, as well as the interplay between different cell types. Additionally, we further substantiate the potential and necessity of dual-pathway combination therapy as a critical target in MET addicted cancer treatment.

Video Abstract

**Supplementary Information:**

The online version contains supplementary material available at 10.1186/s12964-023-01333-8.

## Introduction

HGF/c-MET constitutes a crucial molecular axis that plays a significant role in growth and development. HGF, known as Hepatocyte Growth Factor, represents the ligand, while c-MET refers to its receptor, a receptor tyrosine kinase. This signaling pathway exerts a significant role, characterized by precise temporal and spatial expression, in facilitating processes such as cell proliferation, migration, differentiation, angiogenesis, and even stem cell self-renewal. These functions are crucial during embryonic development, multi-organ formation, and injury repair [1–3]. However, excessive activation or mutation of the c-MET receptor may lead to uncontrolled cell proliferation, evasion of apoptosis, and epithelial-mesenchymal transition. These abnormal phenomena can drive malignant transformation and dissemination of cancer cells, thereby promoting tumor formation and progression [4–7]. Therefore, the HGF/c-MET signaling pathway has emerged as a crucial field for investigating the mechanisms underlying cancer development and identifying potential therapeutic targets.

The Hedgehog (Hh) pathway represents another signaling pathway that plays a key role in growth, development, and cancer [8]. The Hh pathway participates in embryonic development, organ formation, and tissue repair processes, and it is crucial for maintaining normal cell proliferation, differentiation, and pattern formation [9]. Mutations in this pathway can result in various genetic disorders, including Holoprosencephaly, Greig cephalopolysyndactyly, Pallister–Hall syndrome, and Carpenter syndrome, and are closely associated with the occurrence and progression of multiple cancers [10]. Research indicates that abnormal activation of the Hh pathway in many tumor types can promote cancer cell proliferation, survival, and invasive capabilities, as well as contribute to the formation and maintenance of tumor stem cells. Furthermore, abnormal activation of the Hh pathway is associated with key features of tumors, such as angiogenesis, immune evasion, and drug resistance. Consequently, investigating the regulatory mechanisms of the Hh pathway and developing related therapeutic strategies have become important subjects in cancer research, providing new directions and possibilities for cancer treatment.

Cancer-associated fibroblasts (CAFs) are a key component of the tumor microenvironment (TME) and promote tumor progression by promoting angiogenesis, invasion, and metastasis [11]. The HGF/c-MET axis and Hh pathway play critical roles in the interaction between tumor cells and CAFs. It has been demonstrated that the Hh pathway promotes CAF activation and recruits CAFs to regulate cell differentiation [12]. In turn, activated CAFs can stimulate the HGF/c-MET axis in tumor cells through the secretion of HGF, further enhancing tumor stemness, invasion, and metastasis [13]. However, targeting a single pathway among them only demonstrates moderate efficacy in a limited number of tumors. Prolonged usage inevitably leads to drug resistance [14–17]. Therefore, this review aims to explore the mechanisms underlying the interaction between these two signaling pathways in the context of their interaction with tumor cells and CAFs.

This review aims to provide an overview of the current understanding of the HGF/c-MET axis and Hh pathway in tumor cells and CAFs, as well as their interaction with each other. Additionally, we will explore the potential of simultaneously targeting the HGF/c-MET axis and Hh pathway as a rational approach for cancer therapy. We believe that concurrent targeting of these two pathways holds promise for more effective inhibition of tumor growth and positive impacts on drug resistance and progression-free survival in cancer patients.

## The HGF/c-MET Axis in Cancer

### HGF/c-MET Axis

With the advancement of cancer treatment exploration, the HGF/c-MET axis has emerged as a significant target. The HGF/c-MET pathway has been found to be aberrantly activated in a variety of tumors and plays a key role in various biological processes such as tumor occurrence, proliferation, metastasis, angiogenesis, and stemness. c-MET is a receptor for the HGF, encoded by the *MET* proto-oncogene. It is formed through the hydrolysis of its precursor by furin protease, resulting in a dimeric structure composed of an extracellular α-chain and a transmembrane β-chain linked by disulfide bonds [18]. HGF is secreted in the form of a single-chain precursor (scHGF) and undergoes extracellular proteolytic processing to form the active two-chain form (tcHGF), which consists of an α-heavy chain (69 kDa) and a β-light chain (34 kDa). Both scHGF and tcHGF are capable of binding to c-MET; however, only the processed tcHGF can activate c-MET signal transduction. Recent research has shown that in tcHGF, both the high-affinity α-chain and the low-affinity β-chain contain binding sites for c-MET. The binding of the α-chain alone does not activate the c-MET receptor; instead, it acts as a binding domain for c-MET, facilitating the low-affinity binding of the β-chain, ultimately resulting in c-MET activation [19]. In the context of cancer, abnormal HGF secretion and activation, in conjunction with MET gene mutations that are closely associated with overexpression, amplification, and selective splicing, lead to the aberrant activation of the HGF/c-MET axis. Extensive evidence has documented this dysregulation as a driver of tumorigenesis and progression in various cancers, including renal papillary cell carcinoma (PRCC) [20], head and neck squamous cell carcinoma (HNSCC) [21], and colorectal cancer (CRC) [22]. These effects are mediated through a range of downstream signaling pathways, including but not limited to PI3K/AKT, Ras/MAPK, and Wnt/β-catenin.

### Autocrine and paracrine effects of HGF

During growth and development, c-MET is mainly expressed in epithelial-derived cells, while HGF is mainly expressed in mesenchymal-derived cells as a morphogen and plays important roles in organ formation, cell polarity determination, cell migration, tissue injury repair, maintenance of specialized epithelial tissues and other processes through paracrine and/or autocrine mechanisms [23–25]. In tumor cells, specific MET-related mutations have been identified to enhance catalytic efficiency. However, in vitro studies have indicated that these mutations are insufficient to independently induce cellular transformation. Furthermore, overexpression of HGF has been demonstrated to effectively promote tumorigenesis even within cells expressing wild-type MET at normal levels. This phenomenon has been validated in transgenic mouse models, underscoring the paramount importance of active HGF for the activation of c-MET [26]. While some tumor cells are capable of producing HGF and its splice variants, thereby modulating tumor progression through autocrine and paracrine signaling, stromal-derived HGF remains an indispensable element for the sustained activation of c-MET [27–30]. Moreover, more research suggests that some tumor stroma cells, such as CAFs and tumor-associated macrophages, are the main sources of HGF expression and release for paracrine mechanisms [31]. Although most stromal cells have some limiting ability on tumor cells at the early stage, they ultimately promote malignant tumor growth, invasion, and metastasis. They can cause HGF to be present at high concentrations in the tumor stroma by paracrine secretion, thereby promoting malignant behavior of tumor cells [32, 33].

### Interaction between HGF/c-MET axis and TME

High levels of HGF released by various stromal cells in the TME are crucial participants in the malignant crosstalk between the stroma and primary tumors. HGF is abundantly expressed in a critical TME component known as CAFs, while its receptor, c-MET, is highly expressed in tumor cells [34]. Activated CAFs can expedite the growth of diverse malignancies within the organism [35]. Numerous experimental studies have shown that some malignant tumor cells exhibit invasive potential in vivo, but most do not invade the matrix gel in vitro. It is only when co-cultured with CAFs or when their conditioned medium is incorporated into a collagen gel that cancer cells demonstrate invasive potential on the collagen gel. One of the most important cell factors secreted by CAFs in this context is HGF [36–38]. There is evidence that HGF is one of the key molecules that confer invasive growth potential to tumor cells through tumor-stroma interaction [39]. Recent in vivo experiments have further substantiated this notion. In murine tumor models where *HGF* from hepatic stellate cells (HSC)-derived CAFs or *MET* from tumor cell sources was deleted using Lrat-Cre-transgenic mice, a significant reduction in tumor invasiveness and size was observed [40]. These observations have also been confirmed in various cancer types, including breast cancer [41], colorectal cancer [42] and cholangiocarcinoma [43].

Within the extracellular matrix (ECM), an intricate network of proteins and macromolecules exists. This ECM functions as a biological barrier, shielding tumors from immune system responses and the effects of external therapeutic agents. It also serves as a reservoir for various growth factors. HGF has been demonstrated to tightly bind to multiple ECM proteins, including thrombospondin-1 (TSP-1), fibronectin, laminin, type I collagen, heparan sulfate, proteoglycans, and basement membrane components [39]. Through these associations with the ECM, HGF becomes sequestered within it, forming concentration gradients of signaling molecules in different dynamic locations. These gradients induce tumor cell invasion and migration. Both tumor and stromal cells can secrete proteases that modify the ECM, including matrix metalloproteinases (MMPs), serine proteases, cathepsins, and a disintegrin and metalloproteinases (ADAMs), as well as members of the ADAMTS (a disintegrin and metalloproteinases with thrombospondin motifs) family [44, 45]. These proteases, such as HGFA, matrix enzymes, and MMP-2, not only convert scHGF into its mature form, tcHGF, but also facilitate the release of HGF bound to ECM, leading to autostimulation [46]. Certain growth factors released from the ECM, such as transforming growth factor alpha (TGF-α), can interact with c-MET by activating their epidermal growth factor receptors, triggering signal transduction in the absence of ligands [47]. This forms a complex and dynamic network with cytokines and growth factors.

Additionally, HGF secreted by adjacent CAFs significantly enhances the stemness of tumor cells and regulates their metabolism. Common stemness-associated molecules like CD44 can recruit HGF to the cell membrane, promoting its delivery to c-MET and modulating its activity [48]. Cancer stem cells (CSCs) can produce inducers of HGF, including IL-1β, PDGF, TNF-α, bFGF, and prostaglandin E2 (PGE2). These inducers promote the transformation of mesenchymal stem cells, maintain the phenotype of CAFs, leading to increased release of HGF by CAFs. The high concentration of HGF in the ECM acts on the c-Met receptor of tumor cells, further enhancing the invasive growth of tumors.

## The Hh pathway in cancer

### The Hh pathway

The Hh pathway not only synergizes with HGF/c-Met in epithelial-mesenchymal transition but also plays an important role in regulating the TME as a morphogen [49]. The Hh pathway is highly conserved in evolution and has important effects on embryonic development, tissue repair, and ciliary function [50, 51]. Hh was first discovered in *Drosophila melanogaster*, and it was named "Hh" due to the characteristic spiky appearance caused by mutations at the Hh locus, which resulted in the formation of reversed orientation denticle bands on the mutant's cuticle, resembling the spines of a Hh. In mammals, there are three subtypes of Hh: Sonic Hh (Shh), Indian Hh (Ihh), and Desert Hh (Dhh), which are highly conserved in evolution and play important roles in different parts of the body. Among them, SHH has been studied most extensively in tumors [52].Unlike HGF, which requires extracellular processing to mature, Hh protein is processed intracellularly. After translation, the Hh precursor first undergoes self-catalytic cleavage to form a precursor containing an internal peptide thioester. Then, cholesterol reacts with the thioester at the C-terminus, and the N-terminus is palmitoylated by Hh acyltransferase (HHAT) in mammals (Skinny Hh in Drosophila melanogaster). After undergoing these two important lipid modifications, Hh is tightly anchored to the cell membrane. Subsequently, Dispatched homologue 1 (DISP1) acts on the lipid covalently linked to Hh and packages and releases Hh with the soluble carrier signal-peptide-CUB-domain-and-EGF-like-domain-containing 2 (SCUBE2) [53, 54]. The secreted Hh can generate concentration gradients between tissues, and in the limb bud of vertebrates, the distance can reach 300 μm [55]. In addition, Hh can be bound by Hh-interacting protein (HHIP) to inhibit its function [56]. Patched homologue 1 (PTCH1), another important molecule in the Hh pathway and a member of the resistance-nodulation-division (RND) family, is responsible for specifically receiving the lipid-modified Hh [57].

In the classical Hh signaling pathway, Hh transmits signals by binding to Patched (Ptc), a transmembrane protein with 12 transmembrane domains, in conjunction with its receptor. In Drosophila melanogaster, the protein is referred to as Ptc, while in mammals, there are two types of PTCH, primarily protein PTCH1 and PTCH2. PTCH1 is considered to play a major role in this pathway. In the absence of Hh, PTCH1 inhibits the activation of the Hh pathway by suppressing Smoothened (SMO). This effect is likely achieved through PTCH1-mediated efflux of cholesterol from the cytoplasmic membrane, which is required for SMO activation [58]. When lipid-modified Hh binds to PTCH1, PTCH1 no longer inhibits SMO, which can be phosphorylated and activated by PKA, CK1α, and GRK2. With the assistance of Kif3A and β-arrestin, SMO is translocated to the ciliary membrane, where it promotes the nuclear localization of Gli and activates the pathway by inhibiting PKA, GSK-3β, and CK1-mediated GLI phosphorylation-dependent ubiquitination and degradation. Downstream of the Hh pathway, multiple genes, including *Cyclin D*, *Cyclin E*, *Myc*, *Gli1*, *PTCH*, *BCL2*, *VEGF*, *Fox*, *IL-1β*, *IL-3*, *IL-6* and *TNFα* can be regulated by transcription.

### Paracrine secretion of Hh signals in tumors

As previously mentioned, packaged Hh can be released and studies have shown that there is a significant difference in the diffusion distance between Hh released at the primary cilium tip and Hh released at the basal lateral membrane [50]. Released Hh can act on cells expressing Ptch, thereby activating the Hh pathway. This plays an important role in the interaction between tumor cells and other cells in the TME. Some studies have indicated that even in cases where the Hh pathway is not mutated, Hh ligands produced by epithelial cells, including colon cancer, pancreatic cancer, ovarian cancer, lung cancer, oral cancer, and other cancers, activate the Hh pathway in surrounding stromal cells to create a microenvironment conducive to tumor growth, indirectly promoting tumor growth [59–61]. Moreover, in cancers such as prostate cancer and pancreatic cancer, paracrine secretion plays a much greater role than autocrine secretion [62, 63]. For instance, the Hh pathway inhibitor cyclopamine does not significantly inhibit the growth of the prostate cancer cell line 22Rv1 in vitro, but it demonstrates inhibitory effects in xenograft assays [64, 65]. This observation may suggest that the effects of Hh pathway inhibitors are not limited to tumor cells themselves, but rather, attention should be given to the entire TME. Within tumor cells, the production of more Hh protein occurs through non-canonical activation pathways that are independent of SMO. Cells in the TME, in turn, sustain activation in a Hh-dependent manner originating from tumor cells and it leading to the inhibition of the Hh signal has limited effects on tumor cells but is sensitive to stromal cells [66].

## The role of HGF and Hh loop between cancer cell and CAF

CAFs are one of the most important cells in the TME. In the early stages of tumor formation, various fibroblast types and mesenchymal progenitor cells can be recruited and/or activated by tumor cells to affect the multiple biological behaviors of tumor inflammation, fibrosis, and cancer progression. These similar but unique fibroblast types are collectively referred to as CAFs [67]. Based on their phenotypic features, CAFs are currently primarily classified into two main categories: the myofibroblastic CAFs (myCAFs), which express high levels of α-smooth muscle actin (α-SMA) and fibroblast activation protein (FAP); and the inflammatory CAFs (iCAFs), which exhibit secretory features and regulate inflammation [68]. Although driving oncogenic mutations through hijacked growth and developmental pathways in tumor cells are known and essential for cancer research, almost every type of stromal cell has the ability to support cancer cells in specific circumstances. Therefore, the paracrine and mitogenic signals provided by CAFs potentially influence different types of tumors at almost any stage of tumorigenesis and participate in the progression from abnormal proliferation to invasion, migration, and drug resistance.

**Fig. 1:**
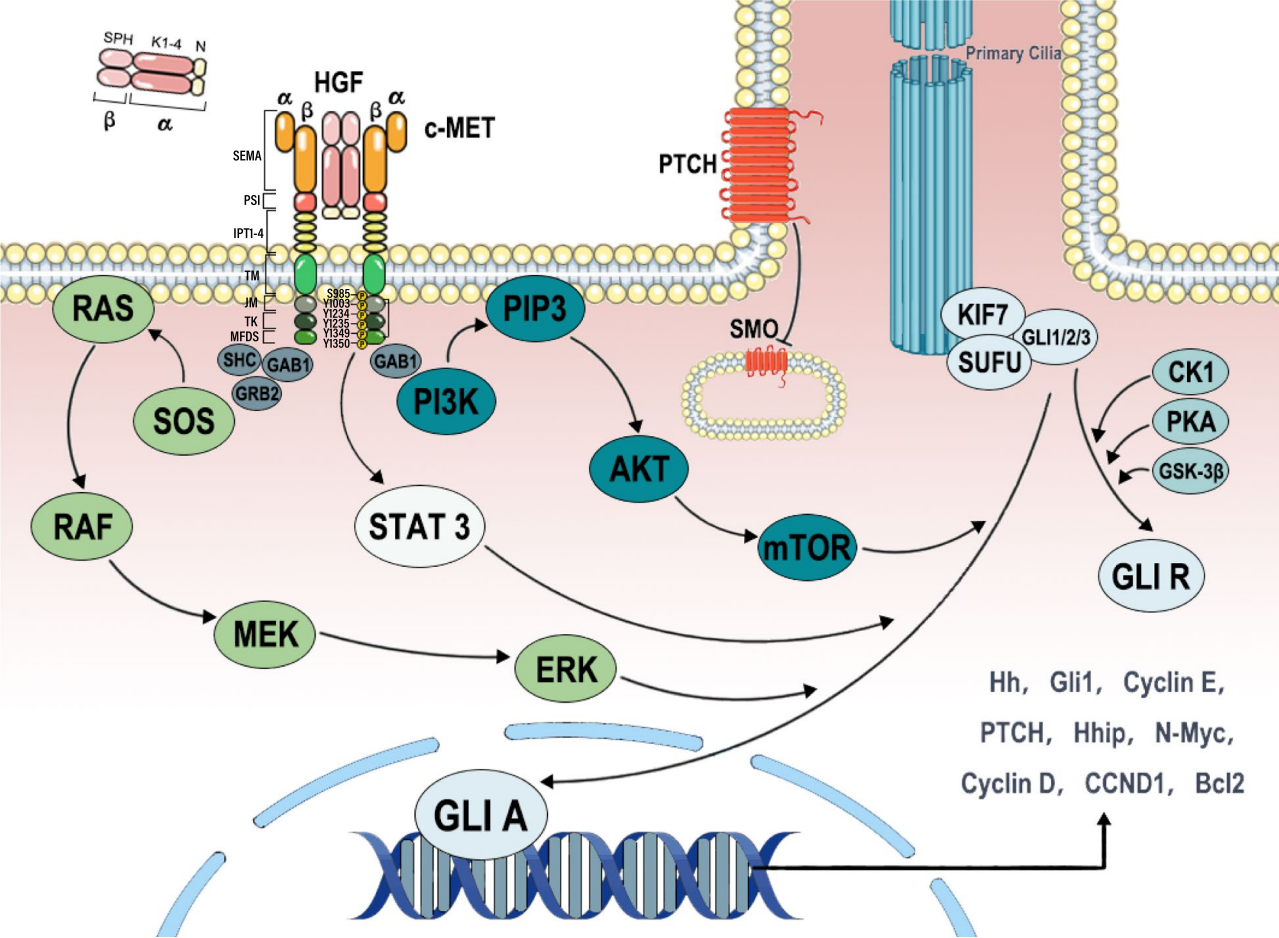
The HGF/c-Met axis activates the Hh pathway through downstream signaling, including MAPK, PI3K/AKT, and STAT3, among others. HGF's kringle domains (K1-K4) structures activate c-Met, while the serine proteinase homology domain (SPH) and N-terminal (N) domains enhance receptor binding. The extracellular portion of c-Met consists of the SEMA domain, the plexin-semaphoring-integrin (PSI) domain, and four immunoglobulin-like regions in plexins and transcription factors (IPT1-4) domain. The intracellular portion includes the juxtamembrane (JM) domain, tyrosine kinase (TK) domain, and C-terminal multifunctional docking site (MFDS). The phosphorylation of S985 and Y1003 in the JM domain leads to ubiquitination-mediated degradation of c-Met, whereas the autophosphorylation of Y1234 and Y1235 in the TK domain upregulates pathway activity by triggering the phosphorylation of Y1349 and Y1356 in the MFDS. The phosphorylation of MFDS primarily serves to recruit downstream proteins

As previously mentioned, CAFs play a crucial role in activating Met signaling in cancer cells by secreting HGF. Similarly, it has been confirmed that HGF expression in CAFs is generally higher than in normal fibroblasts (NFs) and is documented in numerous tumors. These HGF molecules from CAFs activate c-Met in neighboring tumor cells via paracrine signaling in a 2:2 manner. Interestingly, recent research, using structural insights from cryo-electron microscopy, has revealed that a single HGF molecule can bridge two c-MET molecules on opposite sides, leading to their activation and the second HGF molecule further stabilizes this binding and enhances c-MET activation [19]. The two c-MET molecules activated by HGF undergo homodimerization and autophosphorylation on Tyr1234 and Tyr1235, subsequently inducing autophosphorylation of residues Y1349 and Y1356. This activation engages Src homology-2 (SH2) domains, phosphotyrosine binding (PTB) domains, and Met-binding domains (MDB), recruiting various downstream molecules, including GRB1, GRB2, SHC, PI3K, and STAT3, and thereby enhancing Hh pathway activity through multiple downstream signaling pathways [69] (Fig. 1).

The mitogen-activated protein kinase (MAPK) pathway is an important downstream signaling pathway activated by c-Met [70]. After c-MET recruits Grb2 (binding site Y1356), it can activate the Raf/MEK/ERK signaling cascade. Ultimately, not only can GLI1 be activated by ERK2 (at residues S102, S116, and S130) [71], but it can also activate downstream kinases, including MSK1/2 and pp90RSK, to regulate GLI protein activity [72]. And the phosphorylation of Y1356 in c-Met can also activate the downstream PI3K/AKT/mTOR signaling pathway. In esophageal cancer, mTOR can activate GLI1 phosphorylation (at Ser84) through S6K1, leading to the dissociation of GLI1 from SUFU and increasing its transcriptional activity [73]. This concept is supported in studies of prostate cancer and Chondrosarcoma [74, 75]. However, in neuroblastoma research, there was no significant regulatory effect observed, suggesting that tumor heterogeneity may determine differences in signaling pathway activation [76]. Additionally, mTOR can activate the Hh pathway by inhibiting 4EBP1, promoting cell proliferation, which has been confirmed in mouse cerebellum and medulloblastoma [77]. Furthermore, activated AKT not only inactivates GSK-3β but also regulates the Hh and Wnt pathways. AKT can phosphorylate the Ser552 site of β-catenin, increasing its transcriptional activity, inducing the expression of *GLI1* and *GLI2* [78]. STAT3 binds to phosphorylated c-Met, undergoes self-phosphorylation, and translocates to the nucleus, increasing the transcription of genes associated with tumorigenesis [79]. STAT3 not only frequently forms transcriptional complexes with GLI1 and GLI2, binding to their zinc finger domains to promote transcription, but it can also directly enhance the expression of *GLI1* in chronic lymphocytic leukemia cells [80]. Furthermore, recent research has shown that STAT3 is also essential for SMO-dependent signaling in medulloblastoma [81].

When the Hh pathway is excessively activated, downstream genes, including *Hh*, *IL-1β*, *IL-6*, *TNF-α*, *bFGF*, and *FOXF1*, are directly or indirectly transcriptionally activated. This leads to increased expression of CAFs and HGF through paracrine signaling. Notably, FOXF1 can also act as a transcriptional activator for HGF, further promoting its transcription [82]. A comparison of Hh pathway activation in CAFs derived from oral squamous cell carcinoma patients with normal oral fibroblasts reveals that SHH secreted by tumor cells is a key activator of the Hh pathway in CAFs, while in normal oral fibroblasts, although SHH and GLI1 are expressed, the pathway remains relatively inactive [59]. Some studies have demonstrated that paracrine Hh signaling can drive the differentiation of CAFs towards the myCAFs phenotype [33, 40]. The myCAFs produce a significant amount of ECM, resulting in a highly cross-linked ECM that forms a physical barrier, compressing vascular tissue and impeding drug delivery. This phenomenon is particularly prominent in cancers such as pancreatic cancer, cholangiocarcinoma, liver cancer, and head and neck squamous cell carcinoma. Moreover, the dense ECM leads to nutrient depletion and a hypoxic environment, hindering immune cell activation. The hypoxic environment can also induce the overexpression of the MET proto-oncogene and the activation of c-MET, amplifying the HGF signaling pathway and activating the transcription of hypoxia-inducible genes, ultimately increasing the stemness of the tumor. Additionally, Hh pathway-activated tumor cells can rely on downstream inducers of MMPs, including MMP2 and MMP9, to degrade collagen IV, collagen VII, and glycoproteins, thereby modifying the ECM and releasing HGF bound to the matrix. This promotes tumor invasion and utilizes ECM as a nutrient source. When the Hh pathway is inhibited, it leads to a reduction in myCAFs and an increase in iCAFs. It has been reported that iCAFs are the main subgroup secreting HGF, further promoting the activation of Hh within tumor cells to compensate for the inhibitory effects of Hh inhibitors, ultimately increasing the tumor's drug resistance [83].

**Fig. 2:**
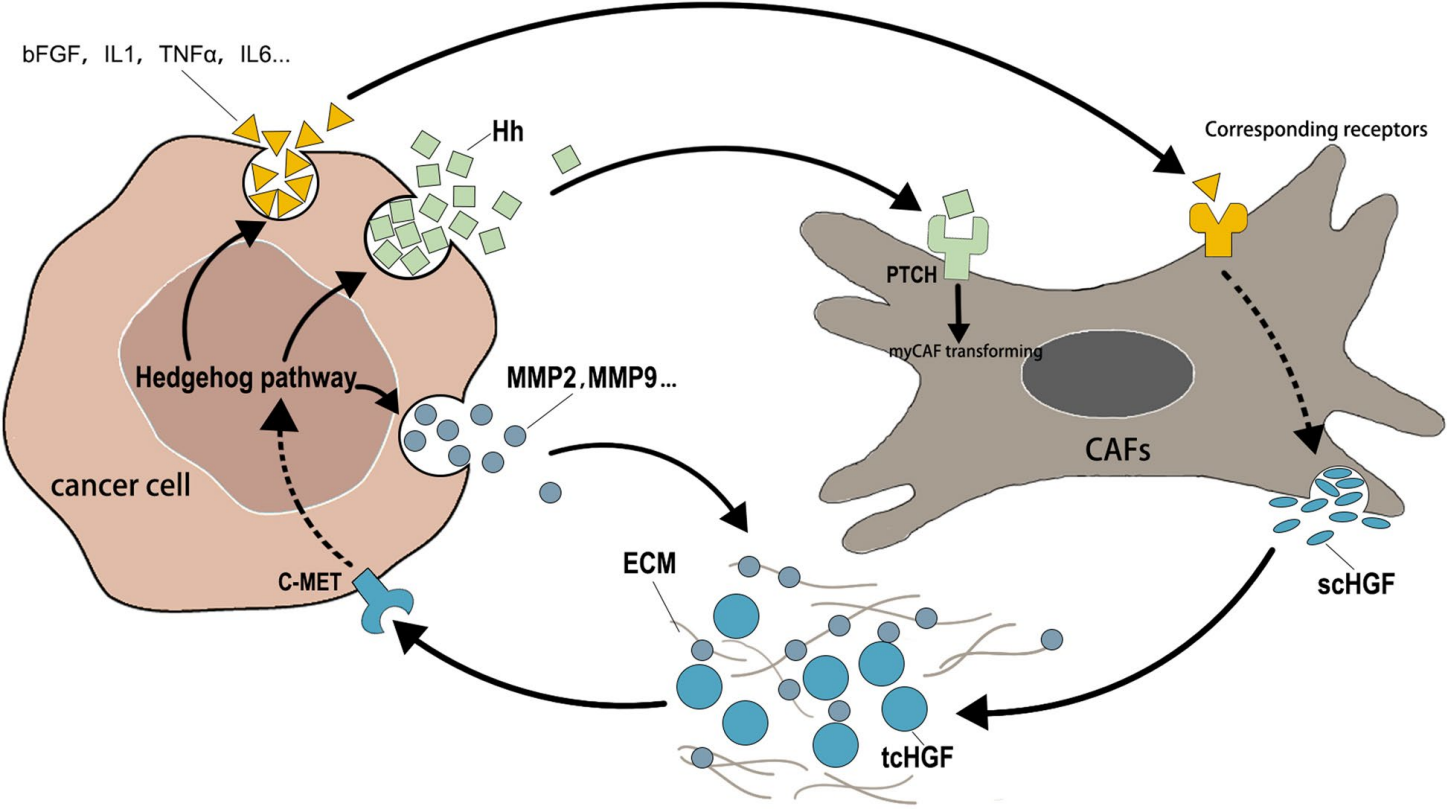
HGF and Hh loop between cancer cell and CAF

The Warburg effect in tumors leads to metabolic changes in tumor cells and alters the TME by producing lactate. HGF and activated myofibroblast-like CAFs (myCAFs) also contribute to this metabolic shift. HGF can enhance the expression of glucose transporters GLUT1 and GLUT4 in several cancers, increasing glycolysis and nutrient consumption [21, 27, 84]. Additionally, the extensive ECM synthesized by myCAFs contributes to hypoxia and lactate production. In this TME, the accumulation of lactate can both increase the expression of HGF in CAFs in an NF-κB-dependent manner and modify the ECM by inducing MMP expression through the ERK/p90RSK pathway in tumor cells [85, 86]. Lactate can also activate signaling pathways such as IL-6/STAT3 and Wnt/β-catenin [87], as mentioned earlier, ultimately leading to the activation of the Hh pathway, creating a vicious cycle. Furthermore, in a study, the authors observed compensatory activation of another pathway in a pancreatic ductal adenocarcinoma mouse model when they inhibited the HGF/C-MET and Hh pathways. This further confirms the conclusion regarding the malignant crosstalk between these two pathways [88] (Fig. 2).

The recruitment of CAFs by tumors involves the secretion of HGF, which in turn activates downstream signal transduction through C-MET within tumor cells. This cascade ultimately leads to the activation of the Hh signaling pathway. Downstream of the Hh pathway, on one hand, there is an induction of Hh, IL-1β, IL-6, TNF-α, bFGF, and FOXF1, promoting the generation of HGF within CAFs. On the other hand, intercellular transmission of Hh can also activate the Hh pathway within CAFs, thereby facilitating their transformation into Myofibroblast-like CAFs (MyCAFs). This transformation results in increased extracellular matrix (ECM) production, induction of stemness, promoting invasiveness, and creating a barrier against immune cell cytotoxicity and drug toxicity. Furthermore, following the activation of the Hh pathway, downstream events include the production of Matrix Metalloproteinases (MMPs) which modify the ECM.

## The clinical status of singular inhibition targeting the HGF/c-MET axis and the Hh pathway

As mentioned earlier, the activation of the HGF/c-MET axis has been reported in various malignant tumors, regardless of whether there is a change in the *MET* genome. This activation is significantly correlated with the tumor's stemness and drug resistance [89]. Currently, c-MET inhibitors are mainly used in non-small cell lung cancer (NSCLC), small cell lung cancer (SCLC), papillary renal cell carcinoma (PRCC), gastric cancer (GC), breast cancer (BC), hepatocellular carcinoma, melanoma, and glioblastoma multiforme (GBM), all of which have a high frequency of *MET*-related mutations (especially the exon 14 skipping alterations (*MET*ex14) and amplification) [90–93]. Targeted therapy against HGF/C-MET currently includes selective c-MET tyrosine kinase inhibitors (TKIs), non-selective c-MET TKIs, anti-c-MET monoclonal antibodies, and anti-HGF antibodies. While many clinical trials targeting HGF/C-MET axis are ongoing or have been completed, most results have not demonstrated complete tumor regression and definitive evidence of target engagement. Furthermore, moderate benefits have been observed for patients receiving C-MET inhibitors in current clinical trials, and progression of cancer and target resistance are inevitable with prolonged treatment [69].

Currently, the widely used Hh pathway inhibitors can be primarily classified into two categories. One category includes inhibitors that target SMO (Smoothened), a key component of the Hh pathway. This group examples include cyclopamine (which inhibits Hh signal transduction by binding to the pocket within the extracellular and transmembrane domains of SMO), TPB15 [94], vismodegib, saridegib, and Sonidegib. Compared to cyclopamine, which had limited success in clinical translation, the latter three drugs have shown more stability and have entered clinical practice or received regulatory approval for market release [95]. In addition, as mentioned earlier, sterols are necessary for SMO activation, and some drugs that block intracellular cholesterol synthesis, such as statins and itraconazole, are used to prevent SMO activation [96]. Due to the occurrence of resistance caused by SMO site mutation after long-term targeting, as well as non-classical pathway-mediated GLI activation, the second type of drugs targeting the Hh pathway are GLI transcription factor inhibitors downstream of the pathway, examples including arsenic trioxide (ATO) [97], glabrescione B (GlaB) [98], HPIs [99], JK184 [100], GANT-58, and GANT-61 [64]. However, similar to targeting HGF/c-MET, clinical benefits of targeting the Hh pathway are limited to a few cancers such as BCC and MB. In addition, clinical trials of combining vismodegib with gemcitabine showed that although Hh signal activity was significantly reduced, the addition of vismodegib did not increase the overall remission rate or prolong patient survival time [101]. These consistent failures indicate the complexity of the Hh signal response and its different roles in cancer that the compensatory mechanisms and complexity of the Hh signaling pathway, as a highly conserved signaling network, as well as its role in different cancers, need to be further explored.

Therefore, targeting the HGF/C-MET axis or the Hh pathway alone as a strategy to combat cancer stemness and drug resistance appears to be insufficient. Considering the complex cellular interactions described earlier and the compensatory nature of these two pathways, combination therapy targeting both pathways may yield better results.

## The clinical impact both Hh pathway and HGF/c-MET axis in met mutation cancers

Undoubtedly, targeting both HGF/c-MET and Hh pathways may produce a more desirable effect, considering the interplay between these two pathways in tumor cells and CAFs, as well as the cross-talk and autocrine mechanisms within cells.

Due to the inevitable development of resistance in non-small cell lung cancer (NSCLC) with TKI treatment, the high frequency of activation of the Hh pathway and c-MET signaling initially drew attention to the dual inhibition of c-MET and SMO. Using an "in silico drug repurposing" and structural analysis approach, glesatinib and foretinib were identified as compounds capable of simultaneously blocking c-MET and SMO. This was validated through cell experiments and xenograft tumor models in TKI-resistant NSCLC [102]. Subsequent research also demonstrated the continued effectiveness of glesatinib in *MET*ex14 and type I MET inhibitor-resistant tumor models and patients. Notably, it remained effective in a patient with *MET*ex14-positive and *MET* Y1230H mutation after relapse following crizotinib treatment [103]. In Phase I clinical trials, glesatinib as a monotherapy achieved an objective response rate (ORR) of 30.0% in the MET-activating mutation population in NSCLC and advanced solid tumors [104]. In another clinical trial for advanced solid tumors, the combination of glesatinib with Erlotinib or Docetaxel achieved an ORR of 1.8% and 12.0%, respectively [105]. In a subsequent Phase II trial for advanced or metastatic NSCLC patients treated with glesatinib as monotherapy, the MET activating mutations in tumor tissue group had a progression-free survival (PFS) of 3.95 months and an ORR of 10.7%, while the *MET* gene amplifications in tumor tissue group had a PFS of 4.84 months and an ORR of 15% (NCT02544633). Similarly, in a Phase I/II multicenter study for advanced hepatocellular carcinoma patients, foretinib showed a PFS of 4.2 months and an ORR of 22.9% [106]. In a Phase II clinical trial for papillary renal cell carcinoma, foretinib demonstrated a PFS of 9.3 months and an ORR of 13.5% [107]. In a Phase II study for patients with triple-negative recurrent or metastatic breast cancer, the overall partial response rate plus stable disease rate was 38%, with median durations of response of 4.4 months and 5.4 months [108]. These results indicate that dual inhibition of c-MET and SMO shows moderate efficacy in unselected advanced solid tumor patients and promising efficacy in late-stage patients with MET mutations.

## Conclusion

In current oncological research, the focus of targeted therapies against CSCs primarily centers around several signaling pathways, including Wnt, Hh, Notch, Hippo, and those closely associated with them, such as NF-κB, MAPK, PI3K, and EGFR [109]. Treatment strategies predominantly aim to inhibit the most relevant pathways within specific cancer types or target appropriate molecular entities within heterogeneous tumor cell populations [110]. Despite the long-standing recognition of these pathways' significance in tumorigenesis and the increasing insights derived from single-cell studies, which uncover subtypes of CAFs within the TME, as well as more detailed descriptions of their involvement in stemness and drug resistance mediated by pathways like HGF/c-MET and Hh, there remains a scarcity of targeted therapeutics directed specifically at distinct CAF populations. Moreover, as of the present, no publicly available clinical trial results have been identified regarding the simultaneous targeting of the Hh pathway and the HGF/c-MET axis. The majority of clinical trials involving Hh pathway inhibitors have primarily focused on assessing their efficacy and integrating these inhibitors into existing chemotherapy regimens, including pyrimidine nucleoside analogs. Nevertheless, these studies often overlook the compensatory signaling that occurs within tumor cells and between tumor cells and other cell types in the TME, leading to suboptimal outcomes upon the introduction of Hh pathway inhibitors in many solid tumors. In contrast, numerous cell-based murine experiments have demonstrated the critical role of interactions between tumor cells and CAFs in tumor heterogeneity, with some clinical trials employing SMO inhibitors and non-specific TKIs yielding favorable outcomes. Furthermore, as previously mentioned, the emergence of specific TKIs targeting SMO and MET further supports this phenomenon, as they both exhibit broad inhibitory effects on advanced solid tumors, including those resistant to conventional therapies. Our research seeks to provide insights into why the singular targeting of the Hh pathway, despite being a critical stemness pathway, yields suboptimal therapeutic efficacy and inevitable resistance when TKIs are employed in isolation. We achieve this by summarizing the intricate crosstalk between the primary c-MET and Hh pathways within tumor cells, the downstream effects of Hh pathway activation on the TME and CAFs, and the role of SHH and HGF serves as a bridge in the context of stromal-tumor interactions. This research has provided a comprehensive analysis of the cross-talk between the primary c-MET and Hh pathways within tumor cells. Furthermore, we have examined the downstream effects of Hh pathway activation on the TME and CAFs. Additionally, we have explored the role of Hh and HGF as mediators in the interaction between tumor cells and the stromal matrix. According to the perspective of intercellular communication and information transmission, our study has addressed the question of why targeting the Hh pathway in isolation results in suboptimal therapeutic efficacy and why the solitary use of TKIs inevitably leads to drug resistance. Importantly, our research offers a deeper mechanistic understanding, particularly for phenotypic studies, and suggests novel directions for multi-targeted approaches in the treatment of cancer.

## Future directions

While the emergence of foretinib and glesatinib and their promising clinical trial results have temporarily addressed the gap in simultaneously inhibiting c-MET and SMO in c-MET-driven tumors, there is still a lack of clinical trial data for more specific and potent agents targeting c-MET and SMO. Clinical trials should be initiated promptly, particularly in tumors exhibiting MET mutations and stromal proliferation. Furthermore, it is important to note that current clinical studies primarily focus on advanced-stage tumors. However, as intra-tumor heterogeneity can develop even within the same tumor type and across different cell populations during disease progression [111], dual-pathway inhibition may yield more favorable outcomes for early-stage MET-driven tumors. Additionally, there is a need for more in-depth research into the intracellular mechanisms of Hh pathway activation in different tumors with MET mutations. Investigating direct interactions among intracellular signaling pathways is crucial for guiding future therapeutic strategies.

Finally, the current emphasis in Hh pathway inhibition predominantly centers on targeting SMO. However, there is a growing body of evidence pointing to mutations that arise as a result of SMO targeting. Many drugs aimed at inhibiting GLI, a key transcription factor in the Hh pathway, face challenges related to toxicity, preventing their clinical translation. Notably, drugs like Itraconazole and ATO, which are already on the market, may induce unavoidable toxicity issues in specific solid tumors due to their distribution. Therefore, it is imperative that more research is directed toward the inhibition of GLI as a transcription factor within the Hh pathway. This avenue of investigation holds the potential to uncover safer and more effective therapeutic strategies. Furthermore, given the frequent mutations observed in the MET gene and the reliance of MET on HGF, it is highly warranted to conduct in-depth research into the development of specific HGF inhibitors tailored to target CAFs. This line of inquiry has the potential to deepen our understanding of the TME and pave the way for innovative, targeted therapies in the field of cancer treatment.

## Data Availability

The data used in this study are all derived from publicly available academic journals, books, or other published sources. All cited data and references can be accessed through their respective publications or databases.
